# Case of Hydrophobia

**Published:** 1826-10

**Authors:** David King


### HYDROPHOBIA.

*Case of Hydrophobia.* By DAVID KING, Esq. (Communicated by Mr. SHAW.)

JULY 22d, 1826.—William Savage, aged sixteen years, of a strong muscular frame and robust constitution, on Monday last (17th,) felt rather dull, and complained of a buzzing noise in his right ear. On the following day this sensation distressed him very much, and some warm milk and oil were put into it, but without affording any relief. However, he was able to walk about during the day, and, after supping on light pudding, he went to bed, without much apparent indisposition; but his mother remarked that he declined drinking any thing. Early in the morning of the 19th, his mother was alarmed by a peculiar noise he made in his throat, as if endeavouring to bring something up. He seemed terrified, and, as soon as he could speak, begged that some of the neighbours might be sent for, as he felt as if he should be suffocated.

About an hour afterwards, he was seen by my assistant, Mr. MIDDLETON. As he was immediately struck with the similarity of the actions and appearance of the patient to the cases of hydrophobia he had seen at the hospitals, he asked the mother if her son had ever been bitten by a dog? The patient, having overheard the conversation, answered immediately, that he had been bit by a strange dog a few weeks ago, and that his brother could tell all about it.

His brother informed us that, on the 10th June last, whilst they were at work, they observed a dog lying in the field, which had "foam" about its mouth; and that his brother, after having pursued it for a few minutes, was slightly bit in the right thumb, whilst endeavouring to get hold of its collar. The part bitten bled a little. The dog was instantly killed, the lad went and bathed in a pond, and no more attention was paid to the circumstance.

I was now requested to visit the patient, when (at seven o'clock) I found him labouring under some difficulty of speech; he sighed frequently, and occasionally sobbed. He was excessively irritable, but his voice was clear and distinct; and he said he felt no pain, but that the buzzing in his right ear continued to distress him very much. He was terrified by the sight of fluids, when brought within a yard or two of him, and was thrown into convulsive spasms even by blowing on his face. He could bear to see fluids poured from one vessel into another, when held at a considerable distance from him; and could look into a mirror, if held at some distance and steadily, but, if moved backwards and forwards, he was much agitated. His countenance was slightly flushed, but there was no livid colour of the lips nor redness of the eyes; his pulse was from ninety-six to one hundred, and rather weak; and his breathing was hurried. He had urgent thirst, and his tongue, which he put out with the utmost facility, was rather white, but moist. He had not the slightest degree of cough, but used occasionally great exertions in attempting to bring up something, which he said made him feel as if he was choking. His bowels had been regularly open every day, but he complained of great weight at the pit of his stomach.

His hand was now washed, and, upon examination, a small cicatrix was observed on the second joint of the thumb of his right hand, which he and his brother pointed out as the part that had been bitten; but there was no redness nor pain about it; nor was there any appearance of irritation on the hand or arm during the whole of the patient's illness.

As fluids could not be brought near him without exciting great distress, a little lemon-juice, with sugar and water, was beat into a pulp with crumbs of bread, and offered him in a teaspoon; when his peculiar manner of swallowing was remarked. He insisted upon taking the spoon into his own hand, and frequently carried it backwards and forwards, to and from his mouth; then turning his eyes away, he would suddenly thrust it, with its contents, into his mouth, with a sort of snatch, and again instantly and violently withdraw his hand, grasping the spoon, and throwing himself back with a convulsive effort. In this manner, however, he always accomplished the swallowing of the substances given in a pulpy form.

His ear was washed out with warm water, and a little oil was dropped into it. Twenty-four ounces of blood were drawn from his arm in a full stream, which did not, after many hours, show the buffy coat; and 120 drops of Battley's Liquor Opii Sedativus were given him, beat up with crumbs of bread.

When visited two hours after, his irritability was rather increased. He requested that no one should come into the room without first, from the staircase, informing him of his approach; and threatened to strike his nurse if she did not keep hold of his hand, or if she attempted to leave his bedside. His pulse was 120, and weak; the upper part of his neck appeared swelled, as if from the enlargement of the submaxillary glands, but he would allow no one to touch his neck; only putting his hand to the lower part of the larynx, he said, "Here is the place I feel the anguish." A glyster of turpentine, salts, and senna, was administered with great difficulty: it came away in about ten minutes, without producing any motion. A blister was applied to the pit of his stomach; four drops of croton-oil were rubbed on his tongue, and, to make sure that some of this medicine might get down into his stomach, he was prevailed upon to take some crumbs of bread, moistened with lemon-juice and water, which he swallowed with the same difficulties and peculiarities as before.

In about two hours after the use of the croton-oil, he had a large scybalous motion, from which he said he felt relief; but his irritability of temper, and anxiety of countenance, continued nearly the same; and his limbs were convulsed upon every exertion, or upon any noise being made in the room. The pupils were observed to be a little dilated in the fore-part of the day; they became considerably so towards the evening, and contracted only slightly on a candle being brought before them. He had lost upwards of ten ounces of blood by the orifice in his arm, from his convulsive exertions; and he began to spit from his mouth a clear viscid saliva, which was frothy, and hung for several inches suspended from his mouth, till removed by the hand. His father conceived he had assisted him very much in bringing up this phlegm, by placing him on his stomach across his knee. He was placed in a tub, and four pailsful of cold water were thrown over him, which he did not seem to dislike or to dread; but, on the contrary, expressed himself relieved from the convulsive action, which, when he was placed on his feet, made him resemble a person affected with St. Vitus's dance.

Some emphysema were perceived about his neck and the upper part of his shoulders, and a distinct crepitus was perceptible under the pressure of the fingers.

Four drachms of Battley's Liquor Opii Sedativus were given him, mixed up with crumbs of bread, and he was assisted to bed. A strait-waistcoat was rather loosely put over him, so that one attendant only might be necessary. The room was kept perfectly free from noise, and rather dark. About eleven o'clock at night, I left him as above described.

The report which his attendants gave of the patient during the short time he lived after this, was that he continued perfectly sensible and quiet, expressing himself thankful for the good effects he experienced from the means used; and that he spoke of the cold affusion as having relieved his thirst, which he attributed to some of it having got down his throat during its application. He never once attempted to sleep, nor complained of feeling uneasy for the want of repose; but was much inclined to talk, and spoke with confidence of being much better next day.

About four o'clock in the morning, five hours after I parted with him, he said he felt so well that he thought he could eat some bread and butter; and, a small slice being given him, he ate it in the way he had formerly taken the other things, and requested afterwards to have something to drink. Some cold tea was brought him, which when he saw, he hurriedly said that there was too much of it; and, having his head raised a little, whilst stretching out his hand towards the cup, he was seized with one of the convulsive spasms of his neck, and expired; being just thirty hours from the time his mother was first alarmed by the appearance of impending suffocation.

*Examination of the body.*—About twenty-seven hours after death, on inspecting the body, the neck and upper part of the shoulders had an emphysematous appearance and feel, and the crepitus was still distinct. The face had no appearance of convulsive action. Upon dissecting off the integuments of the neck, the cellular membrane was found to be distended with air, and the submaxillary glands to be enlarged.

The brain had no unusual marks of vascularity, and there was no effusion of any kind into any of its cavities.

The larynx and the trachea, throughout their whole length, showed appearances of great inflammation. The œsophagus, immediately behind the larynx, had also some marks of inflammation; but in no other part did it, or the stomach, which contained a small quantity of a yellowish fluid, show the least appearance of disease.

In the chest, there were neither adhesion nor effusion. The right lung was rather of a red colour; and, in the left lung, air, in some places, had escaped into the cellular membrane, and was lodged here and there in small sinuses about the size of crow-quills; but this appearance was to no great extent. The pleura costalis, especially on the right side, was of a red colour.

*Eltham; July*, 1826.

---

*Note by* Mr. Shaw, *relative to the preceding Case.*

Mr. King, after having examined the body, sent the larynx and pharynx, with portions of the trachea and bronchiæ, for my inspection. As the parts were quite fresh, and had not been macerated in water, nor their colour changed by spirits, I had an opportunity of examining them with as much advantage as if I had been present at the dissection. The mucous membrane of the larynx bore marks of having been in the highest state of inflammation, being loaded with vessels, as the conjunctiva is in acute ophthalmia, and with numerous specks of purulent secretion, which at first view seemed to be apthous sores. The upper part of the pharynx was also inflamed, but not nearly to the same degree.

As these appearances did not correspond with what I had seen in the dissection of two patients who died of hydrophobia, but so nearly resembled the effects produced by common acute inflammation of the larynx, I suspected that the disease had not been hydrophobia, but an instance of that violent spasmodic affection consequent on inflammation of the larynx, and which is known to be very similar in its symptoms to those of hydrophobia. Unwilling, however, to rely on my recollection of the appearances presented after hydrophobia, I carried the parts, while still quite fresh, to Dr. P. M. Latham, who I knew had very lately examined a case of this kind. He was of the same opinion with me, that, as far as the appearances of the larynx would enable us to judge, the case had been merely an attack of very acute laryngitis.

At this time I had not received the account of the case, which no person, on perusing Mr. King's statement, can doubt to have been one of hydrophobia.

